# Editorial Comment: Does Surgical Treatment for Benign Prostate Enlargement (BPE)-Related Bladder Outlet Obstruction (BOO) Benefit Patients with Central Nervous System Diseases? A Systematic Review

**DOI:** 10.1590/S1677-5538.IBJU.2025.9908

**Published:** 2025-02-15

**Authors:** Ioannis Charalampous, Ioannis Tsikopoulos, Calypso Mitkani, Michael Samarinas, Yuhong Yuan, Ioannis Vouros, Petros Tsafrakidis, Anastasiadis Anastasios, Anastasia Gkotsi, Vasileios Sakalis

**Affiliations:** 1 Hippokrateion Hospital of Thessaloniki Department of Urology Thessaloniki Greece Department of Urology, Hippokrateion Hospital of Thessaloniki, 54642 Thessaloniki, Greece; 2 General Hospital of Larisa Department of Urology Larisa Greece Department of Urology, General Hospital of Larisa, 41221 Larisa, Greece; 3 Agios Pavlos General Hospital of Thessaloniki Department of Neurology Thessaloniki Greece Department of Neurology, Agios Pavlos General Hospital of Thessaloniki, 55132 Thessaloniki, Greece; 4 McMaster University Cochrane UGPD Group Division of Gastroenterology Hamilton ON Canada Division of Gastroenterology, Cochrane UGPD Group, McMaster University, Medicine Health Sciences Center, Hamilton, ON L8S 4L8, Canada; 5 Germal Oncology Center Limassol Cyprus Germal Oncology Center, Limassol 4108, Cyprus; 6 Innovative Surgical and Urological Research Hub Thessaloniki Greece Innovative Surgical and Urological Research Hub (ISUReH), 54250 Thessaloniki, Greece; 7 Aristotele University of Thessaloniki 1st Department of Urology Thessaloniki Greece 1st Department of Urology, Aristotele University of Thessaloniki, 54635 Thessaloniki, Greece

**J Clin Med. 2024 Sep 30;13(19):5846.**

DOI: 10.3390/jcm13195846 | ACCESS: 39407906

## COMMENT

The risk of bladder outlet obstruction (BOO) related to benign prostate enlargement (BPE) increases with age and concerns all men, even those with central nervous system (CNS) diseases, who can also experience lower urinary tract symptoms (LUTS) secondary to the neurogenic lower urinary tract dysfunction (NLUTD) itself (1). Although prostatic surgery is well known to improve outcomes in BPE-related BOO, its efficacy and safety in patients with CNS diseases generates controversy (2, 3). Charalampous et al. conducted a PRISMA systematic review to evaluate postoperative outcomes after surgery for BPE-Related BOO in men with CNS diseases and NLUTD. The review included 13 studies and involved 1,144 patients stratified in four groups: spinal cord injury, Parkinson's disease, post-cerebrovascular accident, and multiple system atrophy. Transurethral resection of the prostate (TURP) was the most frequently performed de-obstruction procedure, followed by prostatic artery embolism and open prostatectomy. The primary outcomes pointed to a substantial improvement in symptoms, with a success rate of 81.4% in spinal cord injury, 27.1% in Parkinson's disease, and 66.7% in post-cerebrovascular accident populations. Continence status was assessed in six studies, pointing out a high risk of postoperative urinary incontinence, particularly in patients with multiple system atrophy, 60% of whom developed de novo incontinence symptoms. Perioperative complications, such as urinary infections, were more prevalent in spinal cord injury patients, and higher perioperative mortality rates were observed in post-cerebrovascular accident patients. These results emphasize the possible benefits of prostatic surgery in properly selected neuro-urological patients while highlighting the importance of careful preoperative assessments to discriminate BPE-related BOO from other etiologies. However, this review only included retrospective studies with a high risk of bias, as well as significant heterogeneity in patient characteristics, diagnosis of BOO, surgery technique, and reported outcomes. Furthermore, most of the studies had a limited number of subjects. Therefore, well-designed prospective studies with standardized inclusion criteria, surgical techniques, and longer follow-up are still needed to determine the real advantage of this intervention in distinct neuro-urological groups.

## ABBREVIATIONS

BPE: = Benign Prostate Enlargement
BOO: = Bladder Outlet Obstruction
CNS: = Central nervous system
